# Functional Annotation of Ion Channel Structures by Molecular Simulation

**DOI:** 10.1016/j.str.2016.10.005

**Published:** 2016-12-06

**Authors:** Jemma L. Trick, Sivapalan Chelvaniththilan, Gianni Klesse, Prafulla Aryal, E. Jayne Wallace, Stephen J. Tucker, Mark S.P. Sansom

**Affiliations:** 1 Department of Biochemistry, University of Oxford, Oxford OX1 3QU, UK; 2 Clarendon Laboratory, Department of Physics, University of Oxford, Oxford OX1 3PU, UK; 3 OXION Initiative in Ion Channels and Disease, University of Oxford, Oxford, UK; 4 Oxford Nanopore Technologies Ltd., Oxford OX4 4GA, UK

**Keywords:** ion channel, hydrophobic gating, membrane protein, annotation, molecular dynamics

## Abstract

Ion channels play key roles in cell membranes, and recent advances are yielding an increasing number of structures. However, their functional relevance is often unclear and better tools are required for their functional annotation. In sub-nanometer pores such as ion channels, hydrophobic gating has been shown to promote dewetting to produce a functionally closed (i.e., non-conductive) state. Using the serotonin receptor (5-HT_3_R) structure as an example, we demonstrate the use of molecular dynamics to aid the functional annotation of channel structures via simulation of the behavior of water within the pore. Three increasingly complex simulation analyses are described: water equilibrium densities; single-ion free-energy profiles; and computational electrophysiology. All three approaches correctly predict the 5-HT_3_R crystal structure to represent a functionally closed (i.e., non-conductive) state. We also illustrate the application of water equilibrium density simulations to annotate different conformational states of a glycine receptor.

## Highlights

• Hydrophobic gating can close ion channels via dewetting of the central pore
• We functionally annotate channel structures via simulation of water within the pore
• The 5-HT_3_ receptor is shown by simulation to be in a functionally closed state
• Three states of the glycine receptor are compared via their pore water density

## Graphical Abstract

**Figure:**
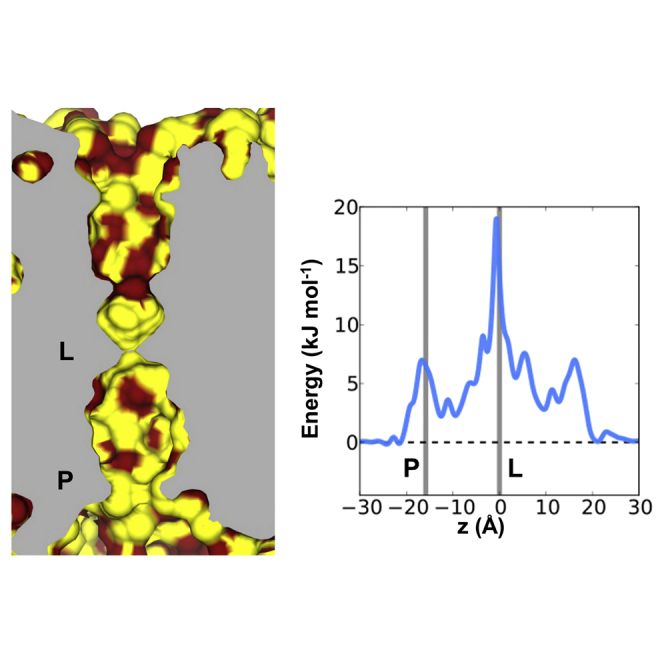

## Teaser

Hydrophobic gating can generate functionally closed ion channel states. Trick et al. show that molecular dynamics simulations can aid functional annotation of channel structures by revealing the behavior of water within transmembrane pores. They describe increasingly complex simulation analyses: water equilibrium densities; single-ion free-energy profiles; and computational electrophysiology.

## Introduction

Ion channels and related membrane proteins play key roles in the physiology of nearly all cells, especially electrically excitable cells within the nervous system. The past decade has seen considerable advances in the structural biology of ion channels; there are currently structures available for >50 different channels and this is likely to rise to >250 by the end of the decade. This interest is driven partly by the considerable pharmacological importance of ion channels, which bind a wide range of drugs from anesthetics (Nury et al., 2011) to antidepressants (Dong et al., 2015). However, it is also complicated by the fact that ion channels are highly dynamic structures that undergo a range of conformational transitions when they move or gate between a functionally closed state (i.e., a state that is impermeant to ions) and an open state that allows selected ions to pass through a central pore at near diffusion-limited rates. Understanding how the specific conformational state of an ion channel relates to its functional properties therefore represents a major challenge facing ion channel structural biologists; there is consequently a pressing need to develop tools that aid this process and allow us to functionally annotate new channel structures as they emerge.

One of the earliest computational tools for functional annotation of channel structures was HOLE (Smart et al., 1996), which enabled visualization and measurement of the dimensions of a pore running through the center of an ion channel structure. This then enabled these pore dimensions to be related to the functional state (open versus closed) of the channel and to the conductance of the channel when open (Smart et al., 1997). A number of related tools for analysis and display of channels and pores in channels have also since been developed; e.g., MOLE (Petrek et al., 2007) and ePOCK (Laurent et al., 2015). However, these methods are generally based on the physical dimensions of pores and cavities within proteins, and do not include explicit calculations of the likelihood of water and/or ions occupying those holes and cavities. This is an important limitation as the character of the pore lining (e.g., hydrophobic versus polar) and its dynamic properties (e.g. rigid versus flexible) play a key role in determining the radius at which a pore becomes permeable to ions (Beckstein and Sansom, 2004).

One of the limitations facing these annotative approaches is that the functional state of a channel (i.e., open versus closed) is not determined by the dimensions and shape of the pore alone. A number of computational and theoretical studies of both model nanopores (Allen et al., 2003, Beckstein et al., 2001, Beckstein et al., 2004, Beckstein and Sansom, 2003, Dzubiella et al., 2004, Hummer et al., 2001, Kalra et al., 2003, Peter and Hummer, 2005, Trick et al., 2014, Vaitheeswaran et al., 2004) and of ion channel structures (Anishkin and Sukharev, 2004, Beckstein and Sansom, 2006, Jensen et al., 2010, Zhu and Hummer, 2012) have indicated that the unusual behavior of water molecules within sub-nanometer radius pores such as those found in membrane ion channels plays a key role in determining their ion permeability.

This has resulted in the concept of hydrophobic gating (Aryal et al., 2014b, Beckstein et al., 2001) whereby expulsion of water or dewetting of a narrow hydrophobic region of a pore results in its functional closure to ion permeation. The degree of dewetting is dependent on the radius, hydrophobicity, and flexibility of the channel (Beckstein and Sansom, 2004). Thus, an ion channel pore might appear sterically wide enough to accommodate water and/or ions, but instead may be functionally closed (i.e., non-conductive to ions) by a central hydrophobic region. If the radius of such a hydrophobic region is less than about 4–5 Å, this can promote dewetting thereby presenting a significant energetic barrier to ion and water permeation. Of course, the absence of dewetting does not necessarily imply that there are no barriers to ion permeation, as the presence of water is not the sole determinant of ion permeability, but it is now clear that the ability of a pore to become hydrated represents a reliable indicator of permeability. This concept of hydrophobic gating has received experimental support from a number of studies of synthetic nanopores (Powell et al., 2011) and ion channels (Aryal et al., 2014a).

It is therefore timely to exploit the concepts underlying hydrophobic gating to develop a more accurate and refined tool for annotation of channel structures. In particular, molecular dynamics (MD) simulations have greatly enhanced our understanding of a wide range of membrane proteins and are now sufficiently robust and computationally inexpensive to allow their routine application to almost any membrane protein structure as soon as it is determined (Stansfeld et al., 2015). This offers the possibility of using MD simulations of water within a novel ion channel structure as a simple computational tool to aid annotation of its functional state. In the current study, we compare the insights yielded by MD simulations of three different levels of complexity as applied to the crystal structure of the serotonin 5-HT_3_ receptor (5-HT_3_R) (Hassaine et al., 2014), a key member of the family of pentameric ligand-gated ion channels (pLGIC) whose functional state was unclear from its structure, but which recent simulation studies have suggested may be functionally closed (Yuan et al., 2016). We then also illustrate further application of the simplest level of this simulation-based annotation approach to three recent structures of the related glycine receptor (GlyR) in different conformational states.

## Results and Discussion

### Initial Simulations of 5-HT_3_R

**Figure 1:**
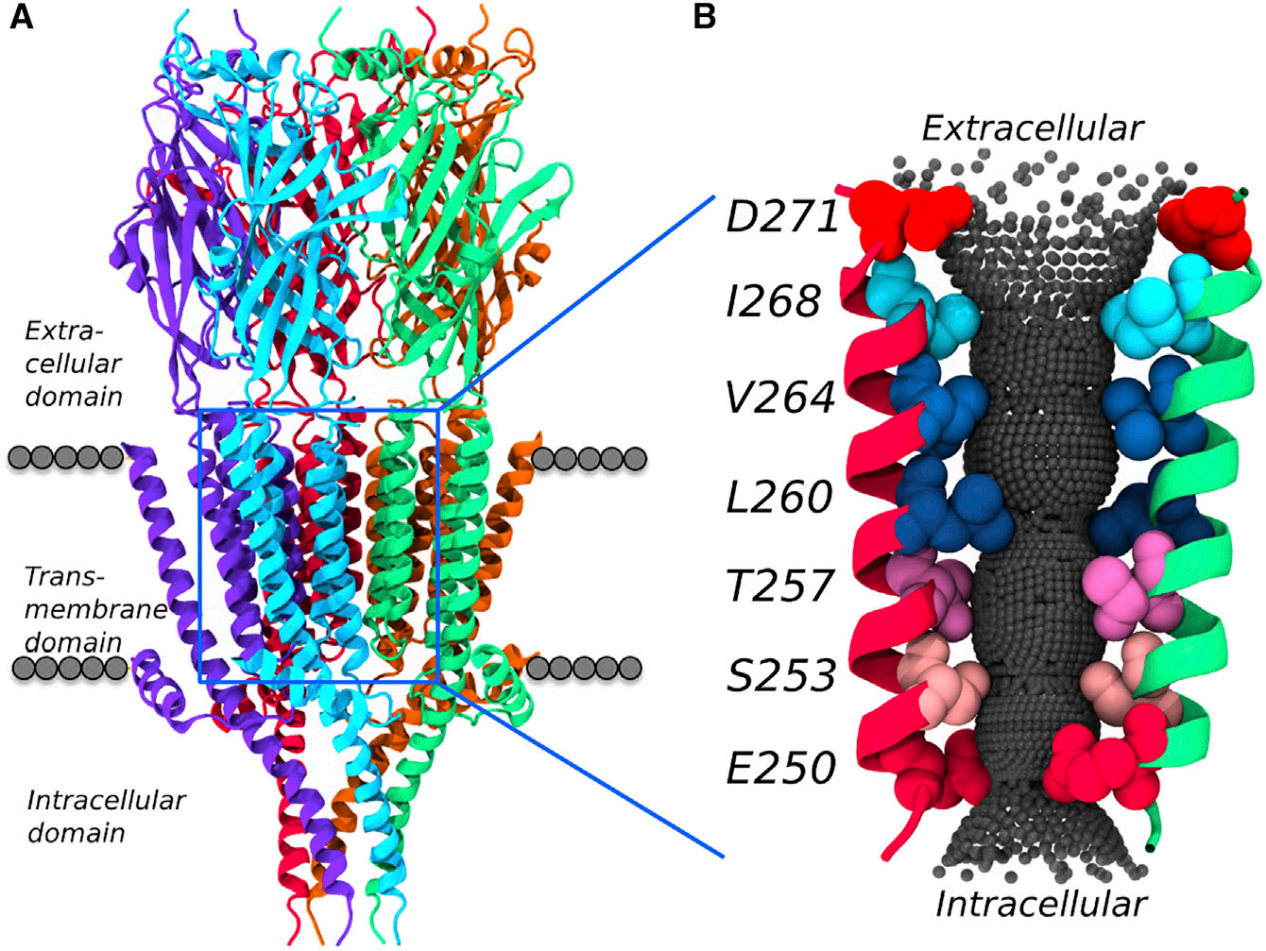
Structure of the 5-HT_3_R, PDB: 4PIR (A) The five subunits of the receptor are shown in different colors, with the approximate location of the lipid bilayer indicated by the gray spheres. (B) Two of the five pore-lining M2 helices are shown along with the pore surface as defined by HOLE (Smart et al., 1996). Key pore-lining side chains are shown in space-filling format, with the hydrophobic gate formed by the V264 and L260 (i.e. L9′) rings in blue.

Although 5-HT_3_R is a canonical member of the pLGIC family of neurotransmitter receptor ion channels (Figure 1A), the functional status of the pore cannot be easily established from simple examination of the structure. The receptor was crystallized in complex with an inhibitory nanobody, which might be expected to stabilize a non-conductive, i.e., closed form of the channel. However, in the paper describing this structure (Hassaine et al., 2014), the uncertainty concerning whether the pore is open or closed is prominently discussed. The dimensions of the central constriction of the pore (formed by a ring of hydrophobic L9′ [≡L260]; Figure 1B) lie in between those expected for an open and a closed channel. In particular, the conformation and orientation of the pore-lining M2 helices resemble those seen in the open GLIC (PDB: 3EAM) and GluCl (PDB: 3RIF) structures, but they differ from those previously seen in, e.g., the closed form of ELIC (PDB: 2VL0). However, the radius of the pore in the vicinity of the L9′ ring (which lies in the center of the bilayer) is ∼2.5 Å, which is in the range where hydrophobic gating might occur (Beckstein et al., 2001, Beckstein and Sansom, 2003, Beckstein and Sansom, 2006). Thus, although the conductive status of this specific conformation is far from clear, it presents an ideal test case to develop and evaluate a hierarchy of MD simulation-based approaches to ion channel annotation.

**Figure 2:**
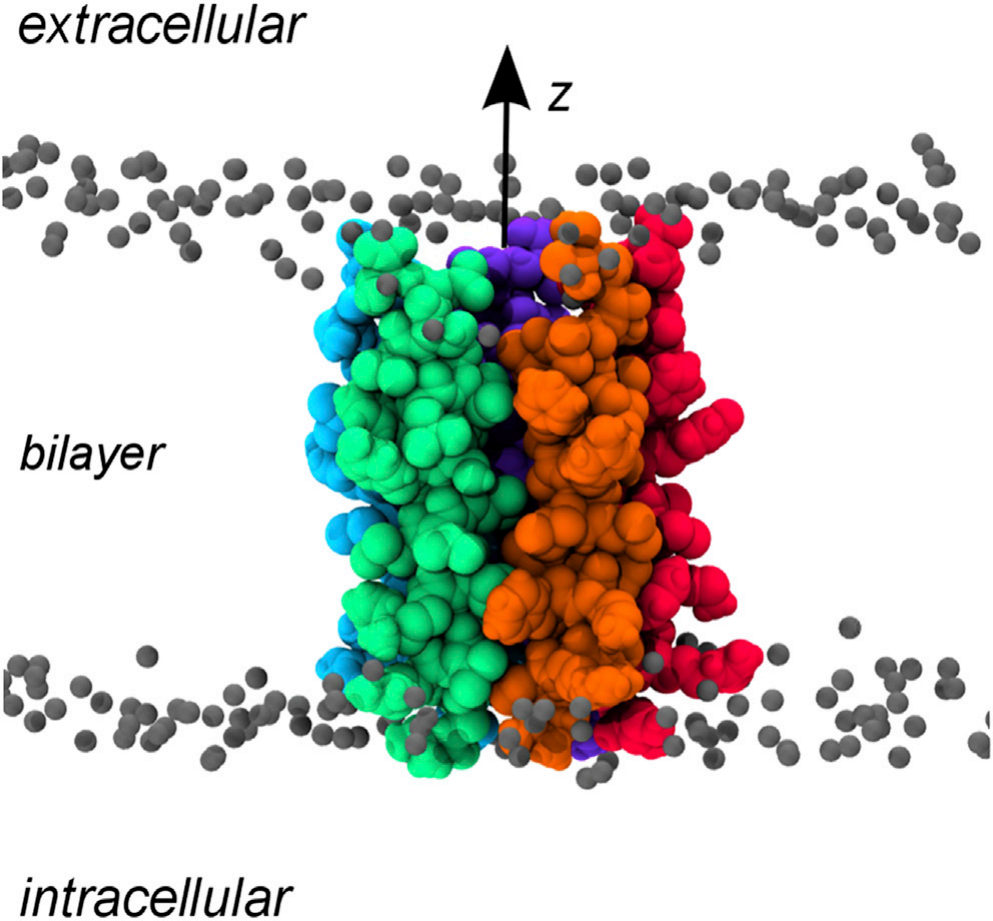
M2 Helix Bundle of 5-HT3R The M2 helix bundle of 5-HT_3_R is shown embedded within a lipid (POPC) bilayer. The arrow indicates the ion permeation pathway and reaction coordinate (*z*) along which the ion and water PMFs are calculated.

Our overall aim is to develop a simulation-based tool for rapid annotation of the functional state of an ion channel structure. It can be computationally demanding to simulate an entire pLGIC in a lipid bilayer (Yoluk et al., 2015), therefore we wished to explore whether more limited simulations based upon the central pore domain of the channel might enable more rapid functional annotation. The proposed hydrophobic gate is located in the center of the bilayer in the pore-lining L9′ position, and so we therefore chose to simulate the core M2 helical bundle embedded in (and spanning) a 1-palmitoyl-2-oleoyl-sn-glycero-3-phosphocholine (POPC) bilayer (Figure 2). To avoid undue conformational drift of the pore domain away from its crystal structure, we applied a degree of conformational restraint. It has been suggested that application of a Gaussian network model (GNM) (Bahar et al., 1997) may be used in such simulations to retain an M2 helix bundle close to the starting (crystal) conformation (see e.g., Cheng and Coalson, 2012). Therefore, we first compared the use of either backbone positional restraints, or a GNM, to restrain bundle geometry in our MD simulations (see Experimental Procedures).

**Figure 3:**
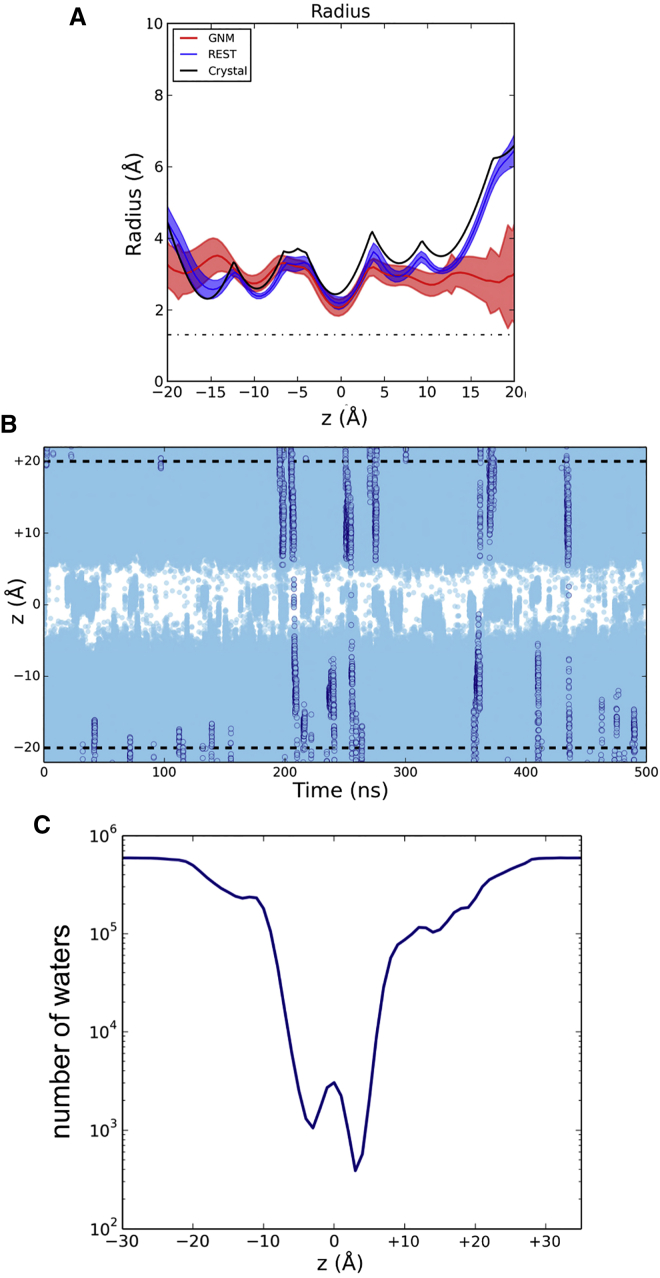
Pore-Radius Profiles and Waters (A) Pore-radius profile in the 5-HT_3_R crystal structure (black line), the positionally restrained simulation (blue line) and for the simulation with a GNM imposed (red line). For the two simulations, the time-averaged radius profile ± SD are shown. The pore is positioned in this and subsequent figures such that the L9′ ring is located at *z* = 0. The dot/dashed line indicates the radius of a water molecule. (B) Positions of water molecules along the *z* axis as a function of time (for the GNM simulation). The two dashed horizontal lines indicate the average phosphate head group position of the lipid bilayer. Each water molecule is represented by a light blue circle. The trajectories of a small number of individual water molecules are illustrated using darker blue circles. Thus, the intermittent white region around z = 0 corresponds to the dewetted L9′ region of the pore. (C) The total number of water molecules across the 500 ns duration (for the GNM simulation) as a function of average position along *z*. Note the use of a logarithmic scale for the vertical axis.

The pore-radius profiles for the crystal structure and for the two simulations (positional restraints versus GNM) were evaluated using HOLE and are compared in Figure 3A. As anticipated, the profiles for the positionally restrained simulation and the crystal structure are very similar, whereas the GNM profile suggests a greater degree of flexibility at either end of the M2 helix bundle. Significantly, however, all three profiles conserved the central constriction (at z ∼ 0 Å) in the vicinity of the ring of L9′ side chains, corresponding to the location of the putative hydrophobic gate. We therefore proceeded to examine the degree of dewetting in this region of the pore.

### Water in the Pore: A First Level of Annotation

At the simplest and most direct level of simulation, one can use the presence/absence of water molecules within a pore as a proxy for ion permeability (Beckstein et al., 2001, Trick et al., 2014). Overall, a low level of water flux was seen through the channel (∼0.1 ns^−1^), comparable with that seen through a model pore with a closed hydrophobic gate (Trick et al., 2014). Comparison of the GNM and M2-restrained simulations revealed very similar levels of water flux in two simulations (0.14 ns^−1^ and 0.2 ns^−1^, respectively) and ions were not observed to enter the pore or cross the central hydrophobic region in either of the 5-HT_3_R pore simulations (not shown).

The wetting/dewetting of the central hydrophobic gate was examined in more detail by tracking the positions of all water molecules along the pore axis as a function of time for the GNM simulation (Figure 3B). From this, it can be seen that only a few water molecules cross the central hydrophobic region and, for more than 50% of the simulation, the pore is completely empty of water molecules (i.e., dewetted, corresponding to a vapor state). Comparable dewetting was seen in a 100 ns simulation with the positional restraints applied to the M2 helix bundle. This may also be quantified in terms of the overall number of water molecules as a function of the position along the pore (z) axis, again averaged over the whole GNM simulation (Figure 3C). A simple Boltzmann inversion (see Experimental Procedures) of the relative density of water within the central hydrophobic region of the pore suggests a barrier of height ∼12 kJ/mol to water permeation in the vicinity of the ring of L9′ side chains.

Thus, annotation based on a short restrained MD simulation of water density within the pore suggests that the crystal structure corresponds to a functionally closed (i.e., ion impermeant) state of the channel. To examine the validity of this conclusion in more detail, we next explored the free-energy landscape for water and ions within the pore.

### Potentials of Mean Force

**Figure 4:**
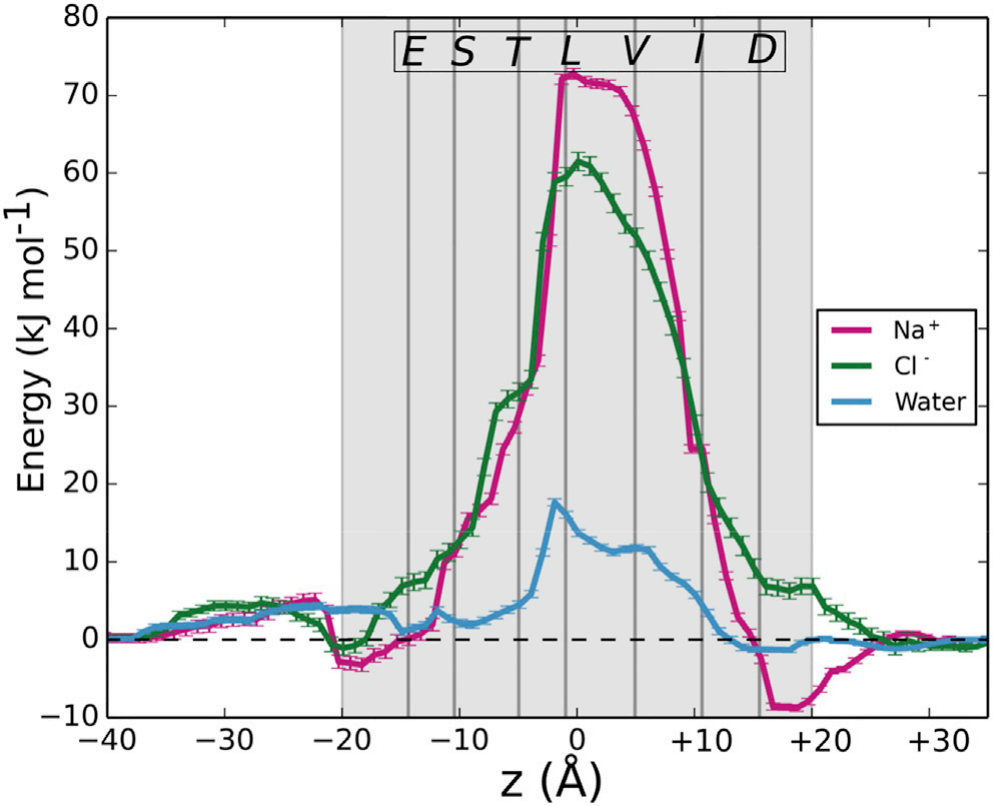
Potentials of Mean Force Potential of mean force (PMF) profiles of single ions (Na^+^ red; Cl^−^ green) or water molecules (blue) as a function of position along the 5-HT_3_R pore (*z*) axis. The gray shading represents the extent of the pore, with secondary vertical lines indicating the approximate positions of the pore-lining side chains. Errors were estimated by bootstrapping.

It is possible to examine a profile of the free energy of a single ion or water as a function of its position along the pore axis by calculation of potentials of mean force (PMF). In such simulations, the ion/water molecule is restrained at successive positions along the *z* axis (while free to move in the *xy* plane), and all other components of the simulation system (protein, ions, water, lipids) are free to move and come to equilibrium. We therefore separately calculated PMFs for a sodium ion, a chloride ion, and for a water molecule as a function of their position along the pore (z) axis (Figure 4). For water, the profile shows a peak in the vicinity of the L9′ ring, with a peak height (relative to bulk water on either side of the pore) of just over +15 kJ/mol. This is consistent with the estimate above calculated from Boltzmann inversion of the water density during the MD simulation (Figure 3C).

For both Na^+^ and Cl^−^ ions, there is a substantive free-energy barrier to permeation (>+60 kJ/mol) centered around the consecutive rings of hydrophobic L9′ and V13′ (≡V264) side chains. Thus, despite the minimum pore radius (∼2 Å) being such that a partially dehydrated ion might pass sterically unimpeded through the pore, it is clear from these estimated free energies of permeation that the pore in this particular structure is functionally closed.

This barrier may also be compared with the hydration numbers of the ions as they move past the hydrophobic constriction. For both Na^+^ and Cl^−^ ions, the inner hydration shell remains largely intact, but the second hydration shell becomes significantly diminished (Figures S1A and S1B). Analysis of the pore-radius profiles for the individual umbrella sampling simulations reveals that even when an Na^+^ ion is restrained within the hydrophobic gate, the local expansion of the pore is minimal (Figure S1C). Comparable dehydration of ions at hydrophobic gates has also been seen in simulations of model nanopores (Richards et al., 2012) and the closed state of the GLIC channel (Zhu and Hummer, 2012).

### Effect of Transmembrane Voltage: Does Electrowetting Occur?

A number of computational (Bratko et al., 2007, Dzubiella et al., 2004, Vaitheeswaran et al., 2004) and experimental (Powell et al., 2011, Smirnov et al., 2011) studies have shown that electrowetting of hydrophobic nanopores may also occur. During this process, the application of a transmembrane voltage may modify the surface tension of water on a hydrophobic surface enough to functionally open a hydrophobic gate. Therefore, given that ion channels are subject to transmembrane potentials of ∼100 mV, we next examined whether electrowetting of the hydrophobic gate within the 5-HT_3_R might also occur.

**Figure 5:**
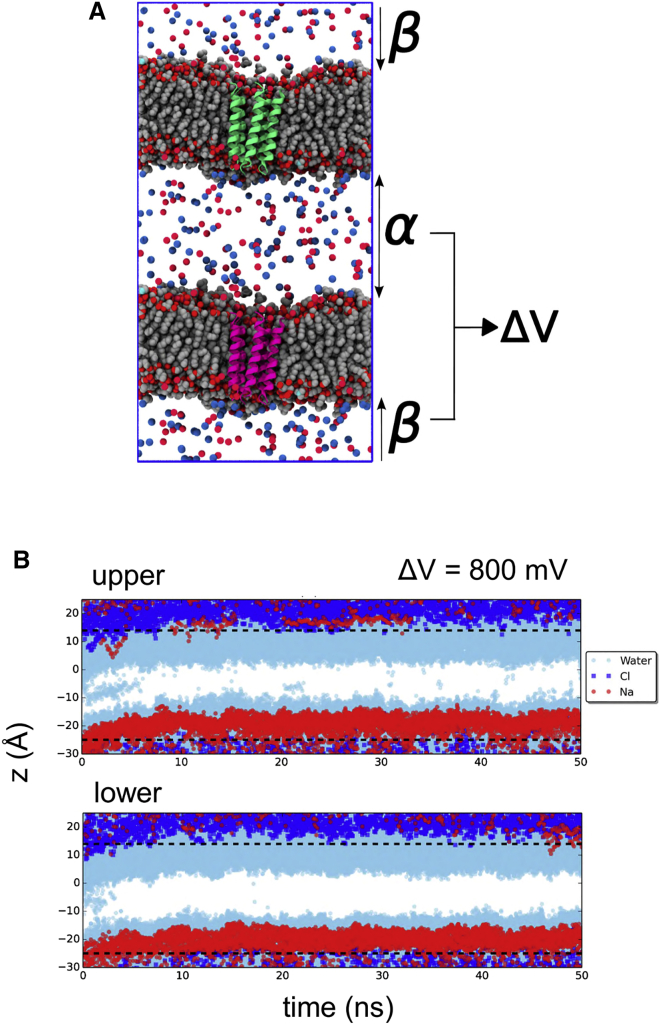
Electrowetting and the 5-HT3R M2 Pore Attempted electrowetting of the 5-HT_3_R M2 pore by computational electrophysiology (CE) (Kutzner et al., 2011). (A) CE simulation setup with two POPC membranes, each of which contains a single bundle of M2 helices (green/pink). Differences in ionic concentrations between the α and β regions generate a voltage difference (ΔV) across the lipid bilayer. (B) The trajectories of water molecules (pale blue; see legend to Figure 3B for details) and of ions (Na^+^ red; Cl^−^ blue) are shown for the two membranes of a CE simulation with a transbilayer voltage difference of ΔV = 800 mV.

There are a number of ways in which MD simulations can be used to mimic transmembrane potentials (see e.g., Melcr et al., 2016 for a recent comparison of different methods). However, the computational electrophysiology (CE) method (Kutzner et al., 2011) is thought to most closely mimic physiological transmembrane potentials by using an explicit ionic concentration gradient (Figure 5A). This allows ion permeation to be simulated directly in the presence of a transmembrane potential and analyzed at atomic resolution (see e.g., Koepfer et al., 2014 for a recent example).

We therefore conducted a set of CE simulations and found that even in the presence of 800 mV across the bilayer (more than five times the physiological maximum voltage difference experienced by most mammalian ion channels), the pore remained dewetted, and no ions entered the pore or crossed the central hydrophobic gate (Figure 5B). It remains possible that electrowetting might still be capable of rupturing this hydrophobic barrier at even higher supra-physiological transmembrane potentials, but these would also be expected to lead to dielectric breakdown of the lipid bilayer (Polak et al., 2013). For a simple model nanopore with a hydrophobic gate (as in Trick et al., 2014) which presented with similar PMF barrier heights for water and ions to that observed for the 5-HT_3_R, both non-physiologically elevated ionic concentration (1 M) and voltage difference (1.2 V) were needed to see electrowetting (J.L.T and M.S.P.S, unpublished data). Therefore, we are confident that electrowetting and subsequent ionic conductance does not occur in the 5-HT_3_R at physiological ionic strength and voltages. Therefore, on the basis of this CE analysis, the pore still remains functionally closed.

### More General Applicability of This Simulation-Based Approach

Having established that MD simulations may be used to functionally annotate the 5-HT_3_R structure, we next explored the more general applicability of this approach. We therefore examined whether the simplest level of simulation, i.e., equilibrium simulations of water within a pore as a proxy for ion permeation, can be used to help annotate other ion channel structures. We decided to undertake this for GlyR, the overall fold of which is the same as for the 5-HT_3_R and for which four structures, three from cryo-electron microscopy (cryo-EM) (Du et al., 2015) and one via X-ray crystallography (Huang et al., 2015) have recently been determined.

**Figure 6:**
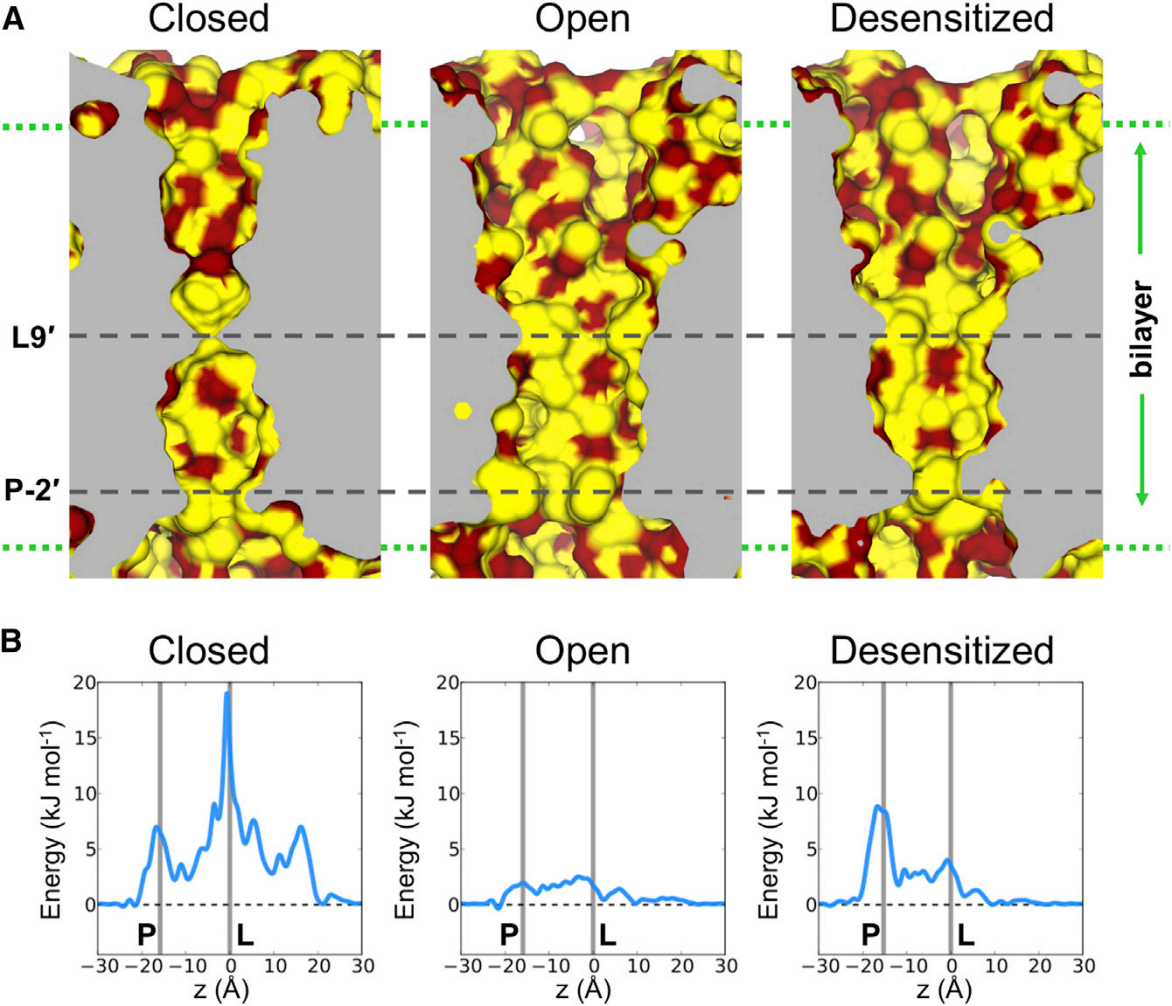
Water within the Zebrafish α1 GlyR Simulations of water within the pore domains of three conformations (closed, PDB: 3JAD; open, PDB: 3JAE; and desensitized, PDB: 3JAF) of the zebrafish α1 GlyR. (A) Longitudinal sections through the center of the pore lumen for the three structures of GlyR. Carbon atoms are colored yellow, and hydrophilic atoms are colored red. The approximate position of the channel within the membrane is marked by dotted lines. The positions of the L9′ (L277) and P-2′ (P266) rings, which constrict the pore, are indicated. (B) Free-energy profiles for water (obtained by Boltzmann inversion of the water density within the pores) for the closed, open, and desensitized states of the GlyR pore. The vertical lines represent the locations on *z* of the L9′ and P-2′ rings.

Three structures of the zebrafish α1 GlyR have been determined by cryo-EM (Du et al., 2015). These three structures have been assigned to functional states on the basis of the known pharmacological properties of bound ligands. Thus, they are suggested to correspond to a closed state (with the antagonist strychnine bound; PDB: 3JAD), an open state (with the agonist glycine bound; PDB: 3JAE), and a desensitized state (with both glycine and ivermectin bound; PDB: 3JAF) of the channel (Figure 6A). We note that that the PDB: 3JAF state has been described as a “potentially desensitized or partially open state” (Du et al., 2015). These three states differ in the orientation of their pore-lining M2 helices. Thus, in the closed state, the narrowest region of the pore corresponds to a ring of L9′ side chains (L277), such that the minimum pore radius is ∼1.5 Å. By contrast, in the open state, the pore radius is increased to ∼5 Å in the vicinity of the L9′ ring, whereas in the desensitized state the M2 helices are tilted such that the narrowest region of the pore (∼2.5 Å) is at its intracellular mouth, corresponding to a ring of P-2′ proline (P266) residues.

**Figure 7:**
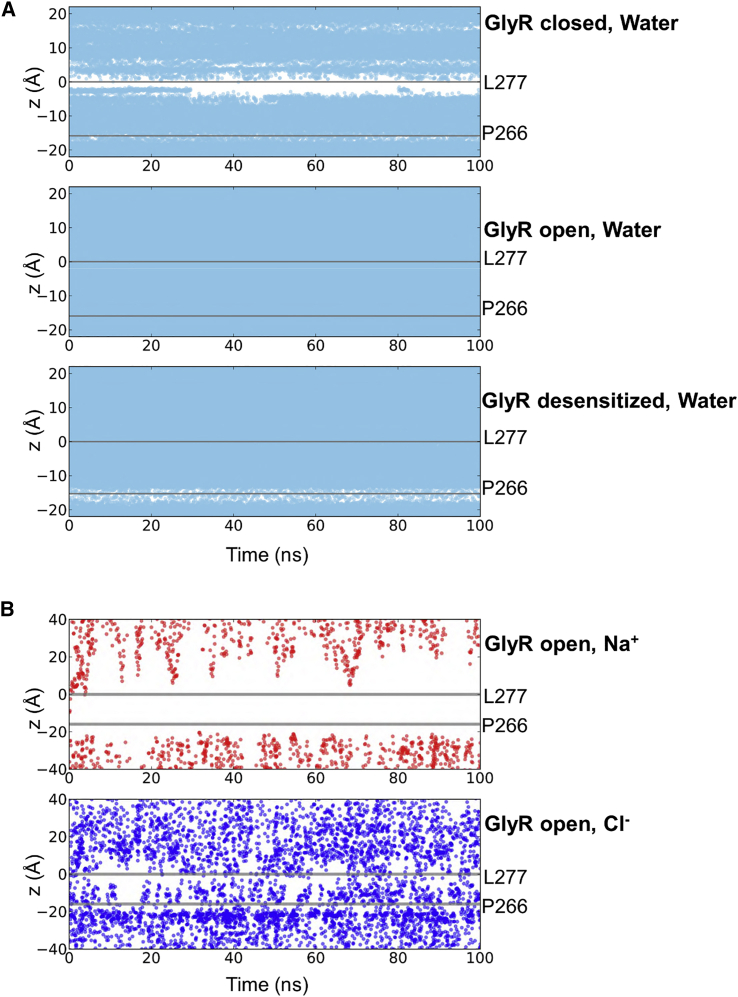
Water and Ions in the GlyR (A) Water positions along the z axis versus time in the pore for the three states of the GlyR. (B) Na^+^ and Cl^−^ ion positions along the z axis versus time in the pore for the open states (PDB: 3JAE) of the GlyR indicating an apparent preference for anions within the pore.

Equilibrium simulations of the entire M1–M4 pore domain (rather than simply the M2 bundle as used in the 5-HT_3_R test case; see above) in a lipid bilayer were performed employing positional restraints on the Cα atoms of the protein (see Experimental Procedures for details) and the water trajectories from 100 ns simulations (Figure 7A) were then analyzed to obtain the average water density as a function of position along the pore (*z*) axis. Boltzmann inversion of the average water densities (Figure 6B) shows that, in the closed form of the channel, there is a barrier of >15 kJ/mol in the vicinity of the L9′ ring (i.e., comparable with that in the closed 5-HT_3_R structure), with a smaller (7 kJ/mol) barrier in the vicinity of the P-2′ ring. In the desensitized structure (which is expected to be functionally closed, i.e., non-conductive), the tilting of the M2 helices reduces the L9′ barrier to 4 kJ/mol (i.e., it wets to a limited extent), but the P-2′ barrier increases to 9 kJ/mol and shows a degree of dewetting. Thus, both states of the channel would be expected to be functionally closed (i.e., non-conductive), with a larger barrier to water (and hence ion) permeation in the closed state of the channel. However, it should be noted that this approach is unlikely to distinguish between subtly different mechanisms of pore closure, e.g., inactivated versus desensitized states, as both are predicted to be non-conductive. As anticipated, the open state of the channel remained fully hydrated throughout the simulation (Figure 7A), and shows small (i.e., ∼RT = 2.6 kJ/mol) barriers close to the L9′ (∼2.5 kJ/mol) and P-2′ (∼4 kJ/mol) side chain rings.

The simulations of the open state also provided some insights into the likely role of the TM pore domain in the mechanism of ion selectivity for GlyR. In these simulations (Figure 7B), we observed that only Cl^−^ ions entered the pore, and that Na^+^ ions were completely excluded. This result is consistent with the anion selectivity of this channel and could provide a basis for further investigation by, e.g., longer CE simulations to give a more quantitative estimate of the selectivity and conductance of the pore (see e.g., Koepfer et al., 2014).

### Robustness of the Simulation Approach

**Figure 8:**
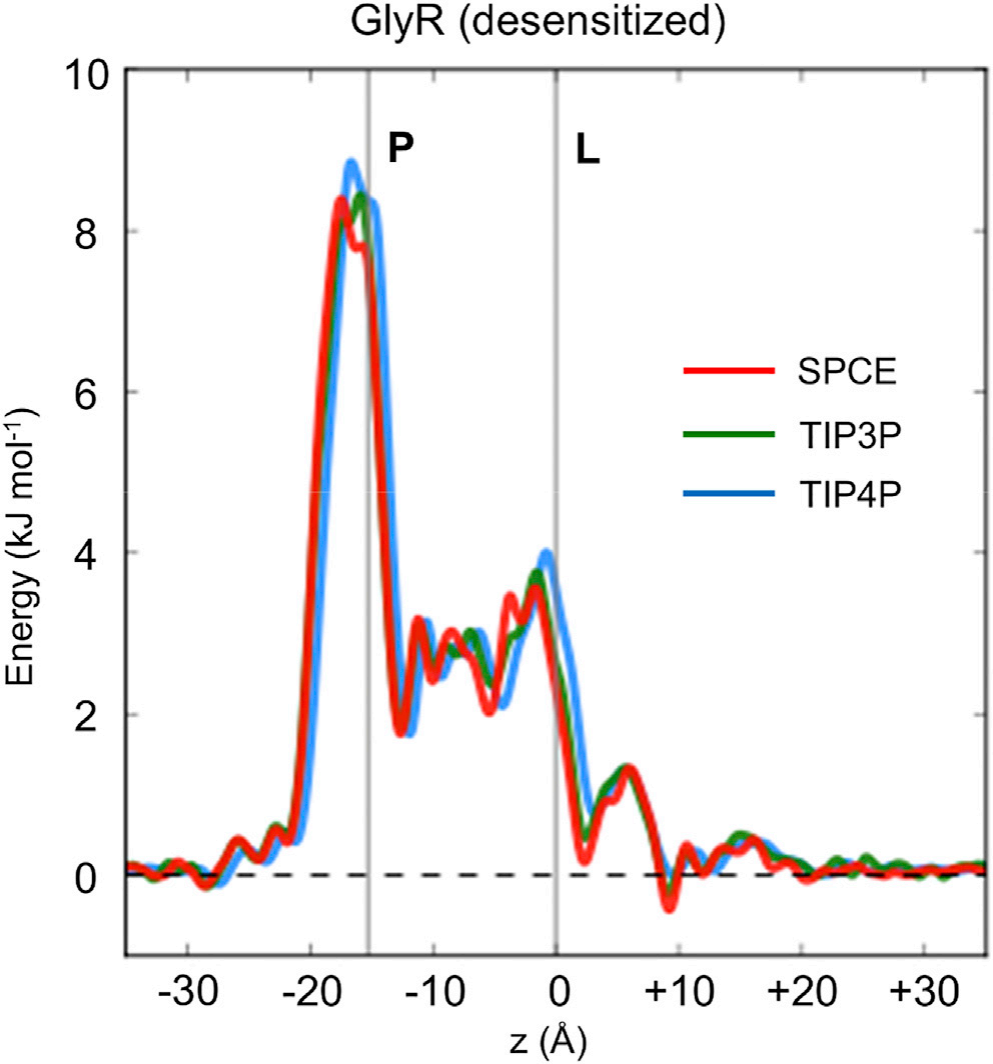
Free-Energy Profiles for Different Water Models in the GlyR Free-energy profiles for water (obtained by Boltzmann inversion of the water density within the pores) for the desensitized state of the GlyR pore. The vertical lines represent the locations on *z* of the P-2′ and L9′ rings. The three profiles shown correspond to different water models used in the simulations: SPCE (red), TIP3P (green), and TIP4P (blue).

To further demonstrate the robustness of this approach, we examined the sensitivity of these simulations to several different types of water model that can be used. We therefore calculated the free-energy profile for the GlyR desensitized state using three widely employed water models (SPCE, TIP3P, and TIP4P) and found that the free-energy profiles for water along the pore were very similar for all three water models investigated (Figure 8). It is possible that the profile might be modified if, e.g., a polarizable water model was also employed, but given the complexities of such water models, further studies on simple model channels are required before polarizable forcefields (e.g., Vanommeslaeghe and MacKerell, 2015) might be routinely applied to a wide range of channel structures.

**Figure 9:**
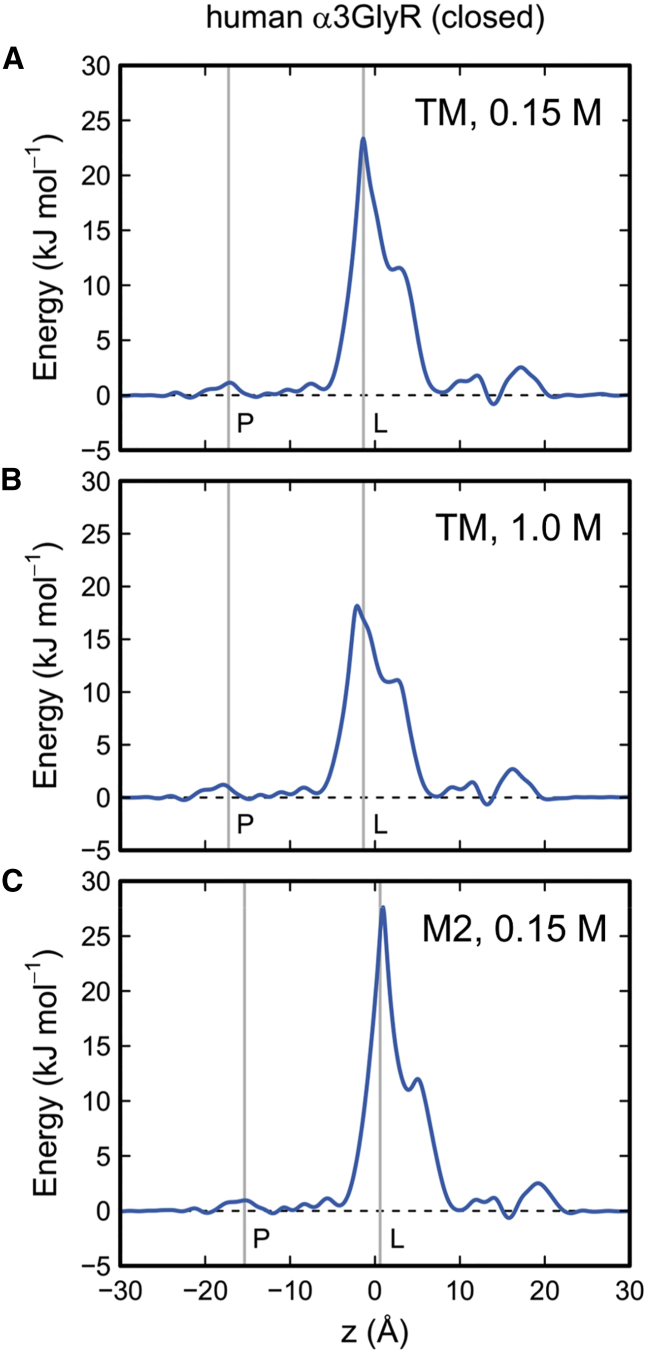
Free-Energy Profile for Water in the Human α3 GlyR (A–C) Free-energy profile for water (obtained by Boltzmann inversion of the water density within the pores) for the closed state (i.e., strychnine bound) of the human α3 GlyR pore (PDB: 5CFB), which was determined at a higher resolution of 3.0 Å. The vertical lines represent the locations on *z* of the P-2′and L9′ rings. Profiles are shown for the TM domain with an ionic concentration of (A) 0.15 M and (B) 1.0 M, and (C) for the pore-lining M2 helix bundle at an ionic concentration of 0.15 M.

We have also examined the effect of resolution of channel structures on the free-energy profile for water within the closed state of the GlyR. For example, the zebrafish closed state (i.e., strychnine bound) structure of the GlyR was determined at 3.9 by cryo-EM (PDB: 3JAD), whereas the structure of the (strychnine bound) closed state of the human α3 GlyR (PDB: 5CFB) has been determined by X-ray diffraction at 3.0 Å (Huang et al., 2015). The sequence of this higher resolution structure is very similar to that of the zebrafish GlyR, differing by only a single amino acid in the pore-lining M2 helices of the two structures. Importantly, we observed that the free-energy profile for water along the pore axis of the α3 GlyR transmembrane domain (Figure 9A) was similar to the corresponding free-energy profile for the α1 GlyR closed state (Figure 6B). In both cases, the central ring of L9′ provides an energetic barrier to water of 20–25 kJ/mol, i.e., a hydrophobic gate. We repeated the simulations of the human α3 GlyR TM domain in the presence of an elevated (1.0 M) salt concentration; this reduced the energetic barrier a little (by just over 5 kJ/mol) but the pore remained functionally closed as judged by the water free-energy profile. We also repeated the simulations with just the pore-lining M2 helix bundle of the human α3 GlyR (with its Cα atoms positionally restrained), instead of the complete TM domain. Again the energetic barrier associated with the L9′ hydrophobic gate was preserved.

On the basis of these comparisons, we may conclude that our simulation approach is robust to differences in structural resolution, the water model employed in the simulations, and small changes in the simulation protocol.

### Conclusions

In this study, we show that a structure of the 5-HT_3_ receptor corresponds to a closed state of the channel. In particular, we have used three successively more detailed levels of simulations to demonstrate: (1) equilibrium simulations of water density within the pore; (2) PMF calculations of free-energy profiles of single ions along the pore; and (3) CE simulations with an ionic concentration gradient (and resultant voltage difference) across the membrane. In addition, we have shown that application of the lowest level of simulation (i.e., equilibrium simulations of water density) to three recently determined structures of another ion channel (GlyR) can also provide insight into their functional status. These studies therefore demonstrate that multiple levels of MD simulations can be used to functionally annotate ion channel structures based upon the principles of hydrophobic gating and that even the simplest level of annotation makes an accurate prediction.

Our use of this new method to identify a hydrophobic gate in the vicinity of the L9′ residue in the 5-HT_3_R is in good agreement with a number of simulations studies that have observed dewetting in this region of the pore and/or comparable energetic barriers to ion permeation in closed-state structures of pLGICs. This has been shown for both the prokaryotic homologs GLIC and ELIC (Cheng et al., 2009, Zhu and Hummer, 2012) and for an invertebrate GluCl receptor channel (Cheng and Coalson, 2012, Yoluk et al., 2015). In these studies, barrier heights in the range 20–80 kJ/mol have been estimated for closed-state pLGICs, which are in agreement with our estimate of 70 kJ/mol for the 5-HT_3_R. An extended (2 nm long) hydrophobic gate has also been suggested to occur in the CorA Mg^2+^ channel, which presents an energy barrier of 190 kJ/mol to permeation when dewetted (Neale et al., 2015). These studies do not of course address the mechanisms of conformational changes underlying channel gating and how this affects permeation; such approaches require more detailed and extended simulation studies (Nury et al., 2010, Yoluk et al., 2015). Indeed, a recent extended microsecond simulation study of the 5-HT_3_R suggests that opening of the pore is associated with a side-chain rotamer change and widening in the region of the hydrophobic gate leading to pore wetting (Yuan et al., 2016). However, we now demonstrate that even short simulations using a simple water model and even structurally reduced models of the pore can be used to provide useful insights into the functional status of these ion channel structures.

In summary, we have demonstrated that a hierarchy of simulation methods enable annotation of the functional state (conductive versus non-conductive) of a variety of ion channel structures. As it focusses on the conductive state of the pore, it is unable to distinguish directly between closed and desensitized/inactivated states. Our approach combines initial screening by equilibrium simulations of water within channel structures with more computationally demanding PMF and CE simulations, providing details of energetic barriers and the effect of applied transbilayer potentials. Importantly, these methods can be readily pipelined to provide an automated web-accessible tool (currently under development) to help with annotation of the ever-increasing number of new ion channel structures that are now beginning to emerge.

## Experimental Procedures

### 5-HT_3_R Simulation System

The transmembrane M2 helices from the X-ray structure of the 5-HT_3_R (PDB: 4PIR; Figure 1) were inserted in a POPC lipid bilayer via a multiscale procedure (Stansfeld and Sansom, 2011), resulting in a simulation system (Figure 2) comprising the pore-lining domain, 320 lipid molecules, ∼25,000 TIP4P (Jorgensen et al., 1983) water molecules, and Na^+^ and Cl^−^ ions at an approximate concentration of 0.15 M.

All simulations were carried out with GROMACS (www.gromacs.org) version 4.5.5 (Berendsen et al., 1995, Hess et al., 2008) with the OPLS United-Atom forcefield (Jorgensen et al., 1996). Equilibrium simulations were conducted with long-range electrostatic interactions treated using the Particle Mesh Ewald method (Darden et al., 1993) with a short-range cutoff of 1 nm, and a Fourier spacing of 0.12 nm. Simulations were performed in the NPT ensemble with the temperature maintained at 310 K with a v-rescale thermostat (Bussi et al., 2007) and a coupling constant τ_T_ = 0.1 ps. Pressure was maintained semi-isotropically using the Parrinello-Rahman algorithm (Parrinello and Rahman, 1981) at 1 bar coupled using τ_P_ = 1 ps. The time step for integration was 2 fs with bonds constrained using the LINCS algorithm (Hess et al., 1997). For the GNM restrained simulations, a Gaussian network was applied (Bahar et al., 1997) to pairs of Cα atoms between 7 and 9 Å apart within the structure, with a force constant of 1,000 kJ mol^−1^ nm^−2^. These simulations took 10 days of CPU time on 32 cores (on 4-core AMD Opteron nodes).

Simulations were analyzed with GROMACS routines, MDAnalysis (Michaud-Agrawal et al., 2011), and locally written code. Molecular graphic images were produced with VMD (Humphrey et al., 1996) and PyMOL (www.pymol.org).

### Umbrella Sampling

The starting system for umbrella sampling simulations was derived from the energy minimized structure mentioned above. Either a single ion or water molecule was placed at successive positions along the central pore axis. Any water molecules or ions overlapping with this ion/molecule were repositioned by energy minimization before simulation. The reaction coordinate thus defined ranged from z +40 to −40 Å with the bilayer center at z = 0 Å, resulting in 80 windows, with a spacing of 1 Å between successive windows. A harmonic biasing potential was applied to the z coordinate of the ion or the O atom of the water molecule with a force constant of 1,000 kJ mol^−1^nm^−2^. Each window was simulated for 2 ns, with no positional or GNM restraints applied to the protein. PMFs were computed using the weighted histogram analysis method (WHAM). PMF profiles were tethered and errors calculated by the bootstrapping procedure within Alan Grossfield's WHAM method (http://membrane.urmc.rochester.edu/content/wham). Convergence of the PMFs was evaluated by calculating the height of the central energetic barrier as a function of time intervals for consecutive 0.1 ns segments extracted from each window (see Figure S2). To obtain a PMF profile for a single species (ion or water) took 5 days of CPU time on 64 cores (on 4-core AMD Opteron nodes).

### Free-Energy Profiles from Water Molecule Distributions

The distribution of water molecules from analysis of an equilibrium simulation may be used to calculate the free energy of a water molecule as a function of its position in the pore. For this, we used the Boltzmann relation between free energy at a given location and the probability of being in that location:

P(z) = (1/Z) exp[−E(z)/kT],

where *P* is the probability, *Z* is the partition function, *E* is the free energy, and *T* is the temperature. The above probability is directly proportional to *n*(*z*), the number density of water molecules, and hence

n(z) = C exp[−E(z)/kT],

where *C* is a constant whose value we set by requiring the value of the free energy to be zero at the two ends of the pore. Inverting the above relation gives

E = −kT ln[n(z)] + kT ln C.

The free energy obtained in this way is plotted against the distance along a pore axis (z).

### CE Simulations

CE simulations were performed using a double bilayer method (Denning and Woolf, 2008, Gurtovenko and Vattulainen, 2005, Sachs et al., 2004) with the implementation of an ion-swap method to enable longer simulations (Kutzner et al., 2011). The overall ionic concentration was 0.15 M, and the transmembrane voltage difference was calculated via the Poisson equation as implemented in the GROMACS tool g_potential, as described in Kutzner et al. (2011). These simulations required 5 days of CPU time for 50 ns on 64 cores (on 4-core AMD Opteron nodes).

### GlyR Simulation Systems

Three starting configurations (Figure 6) for simulations of the zebrafish α1GlyR were generated using a similar procedure to that for the 5-HT_3_R. The transmembrane domains (corresponding to helices M1–M4) of the pentameric GlyR pore in the closed (PDB: 3JAD), open (PDB: 3JAE) and desensitized (PDB: 3JAF) states were embedded in POPC bilayers. Missing atoms were generated using WHAT IF (http://swift.cmbi.ru.nl/whatif/). MD simulations of 100 ns duration were performed as for the 5-HT_3_R (see above). To ensure that the structures did not deviate from the starting cryo-EM structures (because of the missing loops between TM helices), the Cα atoms of the protein were positionally restrained with a force constant of 1,000 kJ mol^−1^ nm^−2^.

The robustness of the estimation of water barrier height obtained from equilibrium simulations to changes in the water model used was explored via simulations of the desensitized state system with TIP3P, TIP4P, and SPCE (see Figure 8). No significant differences in barrier height were observed.

Simulations of the human α3GlyR were initiated using a similar procedure. The transmembrane domain (helices M1–M4) of the pentameric pore in the closed state (PDB: 5CFB; resolution 3.0 Å) was embedded in POPC bilayers, and solvated with TIP4P waters and an ionic concentration of 0.15 M. MD simulations of 100 ns duration were performed with the Cα atoms of the protein positionally restrained.

## Author Contributions

Conceptualization, J.L.T., P.A., S.J.T., M.S.P.S.; Methodology, J.L.T., P.A., M.S.P.S.; Investigation, J.L.T., S.C., G.K., P.A.; Writing – Original Draft, J.L.T., M.S.P.S.; Writing – Review & Editing, J.L.T., P.A., P.A., E.J.W., S.J.T., M.S.P.S.; Funding Acquisition, S.J.T., M.S.P.S.; Resources, M.S.P.S.; Supervision, P.A., S.J.T.., E.J.W., M.S.P.S.

## Supplemental Information

Document S1. Figures S1 and S2

Document S2. Article plus Supplemental Information
