# Supplementary material for: Functional Annotation of Ion Channel Structures by Molecular Simulation
Source: Structure. 2016 Dec 6;24(12):2207–16. doi: 10.1016/j.str.2016.10.005 (PMC5145807; doi:10.1016/j.str.2016.10.005)
Supplement: Document S1. Figures S1 and S2 [file mmc1.pdf]

Structure, Volume *24*

Supplemental Information

Functional Annotation of Ion Channel Structures by Molecular Simulation

Jemma L. Trick, Sivapalan Chelvaniththilan, Gianni Klesse, Prafulla Aryal, E. Jayne Wallace, Stephen J. Tucker, and Mark S.P. Sansom

Supplemental Information for:

Functional Annotation of Ion Channel Structures by Molecular Simulation

Jemma L. Trick, Sivapalan Chelvaniththilan, Prafulla Aryal, Gianni Klesse, E. Jayne Wallace, Stephen J. Tucker & Mark S.P. Sansom

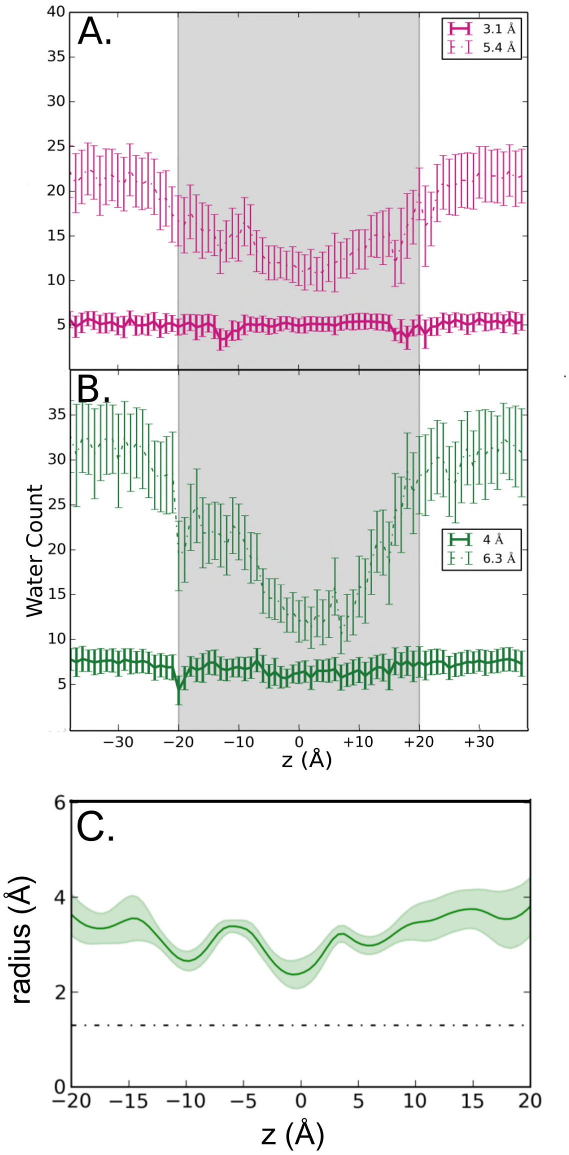

Figure S1, related to Figure 4:

Hydration shells for **A:** Na^+ and **B:** Cl^- ions as a function of position along the pore (z) axis during the respective umbrella sampling simulations the 5-HT_3R. **C:** Pore radius profile from the $z = 0$ Na^+ ion umbrella sampling simulation.

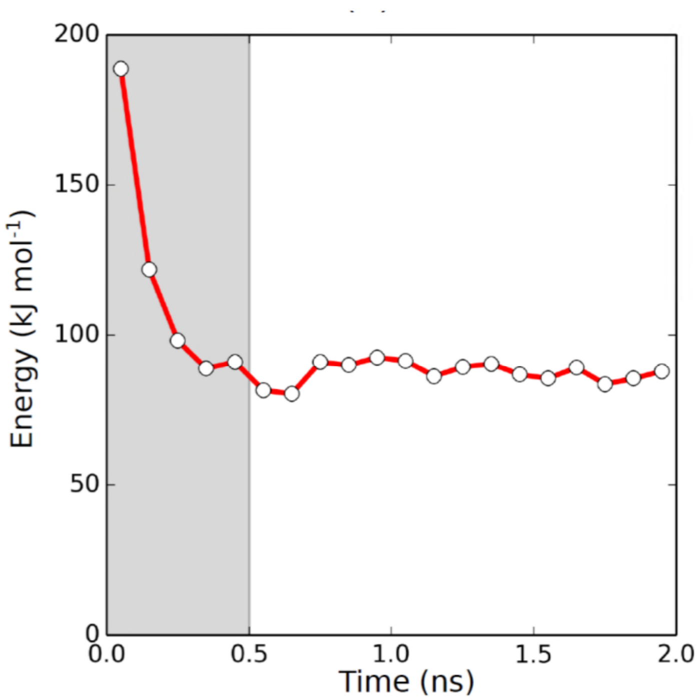

Figure S2, related to Figure 4:

An example of convergence analysis for the Na^+ ion PMF of the 5-HT3R. The graph shows the height of the central free energy barrier (see also Fig. 4) as a function of time, showing the barrier height for successive 0.1 ns intervals along the 2 ns window simulated for the umbrella sampling simulations. The shaded grey region corresponds to the section of the umbrella sampling windows (i.e. the first 0.5 ns) omitted during the final PMF calculations.
